# Differential expression of urinary volatile organic compounds by sex, male reproductive status, and pairing status in the maned wolf (*Chrysocyon brachyurus*)

**DOI:** 10.1371/journal.pone.0256388

**Published:** 2021-08-20

**Authors:** Marieke K. Jones, Thomas B. Huff, Elizabeth W. Freeman, Nucharin Songsasen

**Affiliations:** 1 Center for Species Survival, Smithsonian Conservation Biology Institute, National Zoological Park, Front Royal, Virginia, United States of America; 2 Department of Environmental Science and Policy, George Mason University, Fairfax, Virginia, United States of America; 3 Shared Research Instrumentation Facility, George Mason University, Manassas, Virginia, United States of America; 4 School of Integrative Studies, George Mason University, Fairfax, Virginia, United States of America; University of Hyderabad, INDIA

## Abstract

The maned wolf (*Chrysocyon brachyurus*) is an induced ovulator. Though the mechanism of ovulation induction remains unknown, it is suspected to be urinary chemical signals excreted by males. This study assessed volatile organic compounds (VOCs) in weekly urine samples across 5 months from 13 maned wolves (6 intact males, 1 neutered male, 6 females) with the goal of identifying VOCs that are differentially expressed across sex, reproductive status, and pairing status. Solid-phase microextraction (SPME) and gas chromatography-mass spectrometry (GC-MS) were used to extract and separate VOCs that were identified via spectral matching with authentic standards, with spectral libraries, or with new software that further matches molecular fragment structures with mass spectral peaks. Two VOCs were present across all 317 urine samples: 2,5-dimethyl pyrazine and 2-methyl-6-(1-propenyl)-pyrazine. Fifteen VOCs differed significantly (*Adj*. *P* < 0.001 and |log_2_ fold change| >2.0) between intact males and females. Using partial least squares-discriminant analysis, the compounds with the highest importance to the sex classification were delta-decalactone, delta-dodecalactone, and bis(prenyl) sulfide. Sixty-two VOCs differed between intact males and the neutered male. Important classifier compounds were 3-ethyl 2,5-dimethyl pyrazine, 2-methyl-6-(1-propenyl)-pyrazine, and tetrahydro-2-isopentyl-5-propyl furan. Several VOCs established as important here have been implicated in reproductive communication in other mammals. This study is the most robust examination of differential expression in the maned wolf thus far and provides the most comprehensive analysis of maned wolf urinary VOCs to date, increasing the sample size substantially over previous chemical communication studies in this species. New data analysis software allowed for the identification of compounds in the hormone-producing mevalonate pathway which were previously unreported in maned wolf urine. Several putative semiochemicals were identified as good candidates for behavioral bioassays to determine their role in maned wolf reproduction, and specifically in ovulation induction.

## Introduction

The maned wolf (*Chrysocyon brachyurus*) is a unique member of Canidae (the dog family), endemic to tropical grasslands of South America [1]. The species is listed by the IUCN RedList as “Near Threatened”, with an estimated wild population of 17,000 individuals [2] and a population of <100 in North American zoological institutions. The maned wolf has a large home range [3–5] and is mostly solitary [3–5]. Individuals are of prime reproductive age from 3–8 years old, with rare instances of successful parturition up to 12 years of age [6]. Like other wild canids, the maned wolf is receptive to breeding only once annually, lasting 1–10 days [7–10]. However, because of the solitary nature of the maned wolf, it has evolved an important adaptation to ensure reproductive success. Most canid species studied to date are spontaneous ovulators, meaning that the female does not require a signal prompting ovulation: domestic dog (*Canis familiaris*) [11–13], gray wolf (*Canis lupus*) [14, 15], red wolf (*Canis rufus*) [16], coyote (*Canis latrans*) [17], African wild dog (*Lycaon pictus*) [18, 19], bush dog (*Speothos venaticus*) [20], red fox (*Vulpes vulpes*) [21, 22], and arctic fox (*Alopex lagopus*) [23]. Intriguingly, female maned wolves ovulate only in the presence of a male [7, 24–26]. Because the maned wolf is solitary, this adaptation would improve the likelihood of a successful annual estrous cycle. As of yet the only other canid species presumed to exhibit induced ovulation is the Channel Island fox (*Urocyon littoralis*) [27], though other species of canid have yet to be investigated for this trait.

For the North American population of maned wolves, breeding peaks in November [28]. Female maned wolves housed without a male show baseline progesterone levels through the entire breeding season, indicating a lack of ovulation [7, 24–26]. A maned wolf pair copulated shortly after being reintroduced outside the typical autumn breeding season and gave birth more than two months after the birthing season [10], suggesting that the presence of a male strongly influences the timing of ovulation. Additionally, a female housed singly but sharing a fence line with a male ovulated [25]. In this instance, the female had visual access to the male as well as the ability to contact his urine scent marks deposited on the shared fence line. In the same reproductive season, several other females housed at the same facility with only visual contact to males failed to ovulate [25], suggesting visual stimulus is not sufficient and an olfactory mechanism involving urine underlies this phenomenon. In carnivores, evidence of olfactory signals prompting estrus and ovulation is far less prevalent than in other taxa [29–32]. There does seem to be a male effect in the bush dog, the closest living relative to the maned wolf, where the presence of an adult male decreases the inter-estrus interval of females [20]. However, to our knowledge, the compounds and mechanisms responsible for this effect in carnivores remain completely uninvestigated. The present study begins to fill this gap. In rodents and a marsupial, chemical stimuli can affect reproduction by being aspirated into the vomeronasal organ which has neurons terminating in several nuclei associated with hypothalamic release of GnRH [33–35], so it is possible that a similar mechanism is at work here.

Chemical communication plays an important role in mammalian behavior and reproductive processes for many species [36, 37]. In Canidae, urine is considered to be a more important source of scent signaling than feces [38–41]. Urine scent marking, but not defecation, increases in frequency during the breeding season for several canid species including the maned wolf [8, 38, 39, 41–46]. Maned wolf urine has a very distinctive, pungent odor with scent marks remaining detectable to humans for several weeks [3, 47]. Rates of scent marking do not differ between male and female maned wolves [9]. However the frequency of urine scent marking varies for males across the female reproductive cycle; with the highest frequency during proestrus compared to other stages of the reproductive cycle and higher frequencies for males that successfully breed when compared to unsuccessful individuals [8]. Similarly, females that breed and produce pups scent mark significantly more than those who do not breed [8].

Within Canidae, urinary VOCs have been characterized for the red fox [48–50], coyote [51, 52], domestic dog [53–55], gray wolf [56, 57], African wild dog [39, 40], and maned wolf [47, 58], though differential analysis ascribing putative semiochemical status to certain compounds has only been studied in the gray wolf, a social species. This study is the first robust differential analysis of urinary VOCs for a solitary canid species that relies on chemical communication for reproductive success. Previous analyses of maned wolf urine show that sulfur-containing hemiterpenoids, hemiterpenoid alcohols, and nitrogen-containing pyrazines are main components contributing to this species’ odiferous urine [47]. Despite the fact that the urine of several canids contains the reduced sulfur compound, 3-methyl-1-methylthiobut-3-ene, this compound was not found in the urine of the maned wolf. Instead, an isomer, methyl prenyl sulfide is one of the most abundant urinary volatile organic compound (VOC) in this species [47], further supporting evidence that urinary VOCs are unique to each species. In fact several hemiterpenoid compounds such as geranyl nitrile and methyl prenyl sulfide, thought to be synthesized via the mevalonate pathway, are prevalent in maned wolf urine [47]. Previous studies have also shown that several pyrazine compounds are common in maned wolf urine samples [47, 58]. For the gray wolf, urinary compounds vary with sex of the donor and with season [56]. Furthermore, the administration of testosterone to neutered male gray wolves induces the production of urinary VOCs usually associated with intact males [57], supporting the idea that urinary compounds are correlated with reproductive hormones, and thus, reflect reproductive status.

This study is the first study to analyze the differential expression of VOCs in maned wolf urine by sex, male reproductive status (intact or neutered), and by pairing status within each sex. The primary goal was to identify putative semiochemicals responsible for chemical communication regarding reproduction, which is critical to reproductive success for this solitary species. With a 3-fold increase in sample size over our previous work [59] and a 10-fold sample size increase over other previous studies in this area [47, 58], we describe the abundant VOCs in maned wolf urine and identify several prenyl compounds not yet identified in maned wolf urine. This work lays the foundation for categorizing the compounds responsible for ovulation induction in the maned wolf to better understand this unique reproductive mechanism within Canidae.

## Materials and methods

### Animals

Sample collection procedures were submitted to the Institutional Animal Care and Use Committee of the Smithsonian National Zoo and Conservation Biology Institute and George Mason University but were deemed to be exempt from full review due to the non-invasive nature of the urine sample collection. Eighteen maned wolves were enrolled in the study, but five did not provide regular samples and were excluded from analysis. Thus, thirteen maned wolves (seven males, six females) (Table 1), were sampled for this study from August 1, 2014 to December 31, 2014, surrounding the November peak breeding season. This represents over 15% of the North American population and the largest sample to date for this species. Animal care staff were unable to attribute samples from brothers SB#3192 and SB#3195 to one particular wolf, so each urine sample was from one or potentially both brothers. As such, these brothers were treated as one wolf for analyses. All other samples were from one, known maned wolf only and were not overmarked by other individuals housed within the same enclosure.

**Table 1:** Demographic information for maned wolves that supplied urine samples.

SB#^a^	Age	Sex	Institution^b^	Housed with^c^	Pairing Status^d^	Breeding Information^e^
3231	3	F	Beardsley	SB#3232 (same sex sibling)	Unpaired	N/A
3232	3	F	Beardsley	SB#3231 (same sex sibling)	Unpaired	N/A
2845	8	F	Fossil Rim	*	Unpaired	None seen
3192	3	M	Philadelphia	SB#3195 (same sex sibling)^$^	Unpaired	N/A
3195	3	M	Philadelphia	SB#3192 (same sex sibling)^$^	Unpaired	N/A
2810	8	M	SCBI	Single	Unpaired	N/A
3206	4	M	Fossil Rim	SB#3207 (spayed sister)	Unpaired	N/A
2844	8	M	SCBI	SB#3184	Paired	None seen
3184	4	F	SCBI	SB#2844	Paired	None seen
2660	10	M	WOCC	SB#2945	Paired	Estimated breeding date Oct 8, 2014
2945	8	F	WOCC	SB#2660	Paired	Estimated breeding date Oct 8, 2014
2536	11	F	Buffalo	SB#3014 (neutered male)	N/A	N/A
3014	7	NeutM	Buffalo	SB#2536 (11 yr old female)	N/A	N/A

^a^Studbook Number. Association of Zoos and Aquarium reference number of individual pedigree and demographic history.

^b^Institution

Beardsley: Beardsley Zoo, Bridgeport, CT; Buffalo: Buffalo Zoo, Buffalo, NY; Fossil Rim: Fossil Rim Wildlife Center, Glen Rose, TX; Philadelphia: Philadelphia Zoo, Philadelphia, PA; SCBI: Smithsonian Conservation Biology Institute, Front Royal, VA; WOCC: White Oak Conservation Center, Yulee, FL

^c^Housed with: Details on the housing situation for each individual.

*SB#2845 was housed with SB#3006 (male) and her 3 yearling pups from Aug 1—Sept 9, 2014 then with her 3 yearling pups from Sept 10—Dec 31, 2014.

^$^Urine samples could not be individually attributed between this pair of brothers.

^d^Pairing Status: Unpaired: not housed with a reproductive mate; Paired: housed with a reproductive mate; N/A: excluded from analysis of paired v. unpaired individuals because female was past prime reproductive age and male was neutered.

^e^Breeding Information: Details on breeding are included here. N/A is used in cases where there was no opportunity for breeding to occur due to pairing status.

All animals were housed according to Species Survival Plan recommendations, in an outdoor enclosure with access to indoor den space [6, 10]. Of the six females included in the study, three were not paired with a male (unpaired) (SB#3231, SB#3232, SB#2845), two were paired with a male (SB#3184, SB#2945), and one was excluded from the paired/unpaired analysis (SB#2536) because although she was housed with a male, he was neutered. Of the seven males, four were classified as unpaired (SB#3192, SB#3195, SB#2810, SB#3206) while two were paired (SB#2844, SB#2660), and one neutered male (SB#3014) was excluded from the paired/unpaired analysis. The neutered male (SB#3014) was fully castrated as a standard treatment of congenital unilateral cryptorchidism. The procedure was completed when the individual was 10 months old in October 2007, several years prior to the start of this study. The procedure was not performed for the present study, but afforded an excellent opportunity to compare urinary VOCs from this individual with no testosterone-producing testes with VOCs produced by intact males.

Because the North American maned wolf population is centrally managed by a Species Survival Plan [6], all wolves were fed the same diet of maned wolf chow (Mazuri, Land O’Lakes, Inc., Richmond, IN) supplemented with seasonal fruits and whole prey items (mice, guinea pigs, fish). Diet does alter urinary VOCs [60, 61] but because all individuals were maintained on a similar diet, this is not a concern for the present study. Water was available *ad libitum*. Urine samples (2–15 mL) were collected opportunistically, 1–2 times weekly. Samples were usually collected within a few minutes of elimination (but up to eight hours after) from a washed (water only) stainless steel urine catchment tray hung on the fence or laid on the ground. Rarely, samples were pipetted from the concrete den floor immediately after elimination. Because analyses in the present study target VOCs that are soluble in urine and are based on presence / absence and relative abundance of a VOC, the effect of samples of differing ages should have little impact. Samples were transferred in polypropylene centrifuge tubes (Corning, Inc., Tewksbury, MA) and were frozen at -20°C immediately after collection until processing, which took place March through October 2015.

Overall, 317 urine samples were collected and used for analysis. This corresponds to an average of 26.42 ± 7.00 (SD) samples per individual.

### Sample preparation and GC-MS

The sample preparation and analysis were previously described in detail [59]. Briefly, each urine sample was run in triplicate; three 1 mL aliquots were pipetted into 10 mL clean glass headspace vials along with ACS grade sodium chloride to saturation, and 153 ng of naphthalene-d8 as an internal standard for normalizing peak areas between samples.

Samples were analyzed by headspace solid-phase microextraction (SPME) on a 7890A-5975C gas chromatograph-mass spectrometer (Agilent Technologies, Santa Clara, CA) fitted with a CombiPAL robotic sampling preparation and injection system (Autosampler Guys, Alexandria, VA). The CombiPAL heated and agitated the sample at 37°C for 30 min to equilibrate the sample with the headspace, then for 45 min to equilibrate the headspace with a 1 cm 50/30 μm divinyl benzene-carboxen-poly(dimethylsiloxane) (DVB/CAR/PDMS) stable-flex SPME fiber (Sigma-Aldrich, St. Louis, MO). The SPME fiber and column were heated to the maximum temperature between samples to prevent carry-over. The instrument and autosampler system were controlled using MSD Chemstation software ver E.02.02 (Agilent Technologies, Santa Clara, CA). Chromatographic separation was obtained with a 0.25 mm ID by 30 m long SUPELCOWAX 10 column with a 0.25 μm film (Sigma-Aldrich, St. Louis, MO). The mass spectrometer operated in the full scan mode with a range from 40 to 350 m/z.

### Peak picking and grouping

The raw data files in MassHunter’s.D format were converted into.mzXML format for data processing steps using ProteoWizard’s MSConvert tool [62]. Files from replicates of each sample were processed together through the xcms R package [63] using R ver 3.6.1. The xcms package contains functions to identify ion peaks, group the same ion peaks across replicates, correct retention time drift using the Obiwarp algorithm [64], and then use the corrected retention times to regroup ion peaks across replicates. Next, missing ion peaks were filled in. Then a deconvolution algorithm in the camera R package [65] assigned ion peaks into compounds. Ions with the same peak shapes at the same retention times were attributed to the same group to create a compound made up of component ions. This open-source data analysis pipeline is robust, free of cost, and easily accessible, allowing analysis of hundreds or thousands of samples containing millions of ion peaks on a personal computer. Thus, this same data analysis pipeline could easily extend to other species or areas of VOC research. Ion peak areas were then averaged across the three replicates of each sample, creating one mean value per urine sample for each ion. Within each compound group, only the peak area for the quantitative ion (ion with the largest average area) was retained. This value is representative of the abundance of the VOC in that sample.

### Normalization and scaling

Processed data were analyzed using the web-based platform MetaboAnalyst ver. 4.0 [66]. Quantitative ion peaks were aligned across samples (m/z tolerance = 0.25, RT tolerance = 1 s). Missing data (i.e., VOCs that were not found in a given sample) were replaced with a low value for peak area equal to 1/5 the minimum observed value in the overall dataset [67]. Raw VOC abundances were normalized to the abundance of the internal standard using probabilistic quotient normalization [68] and then generalized log transformed [69] and pareto scaled [70] prior to statistical analysis.

### Abundant VOCs

Presence / absence and raw VOC abundance were analyzed to determine which VOC were found most often and in greatest abundance across the 317 samples. For each group (males, females, neutered male), the mean and standard deviation of the raw abundance were calculated along with the percentage of samples within the group that showed the VOC. The ten most abundant VOC present in at least 70% of the samples for that group are reported.

### Statistical analysis and classification

To determine which VOCs were differentially expressed, the normalized, transformed, and scaled abundance of each VOC was compared across groups using independent samples t-tests. Comparisons were: male versus female, intact versus neutered male, and paired versus unpaired males and females. Raw *P*-values were adjusted for multiple testing based on the false discovery rate (FDR) and were considered significant at an FDR adjusted *P*-value < 0.001. Next, log_2_ fold change was calculated comparing the normalized, transformed, and scaled abundance of each VOC across groups to understand the magnitude of the difference. VOC with an absolute value of the log_2_ fold change > 2.0 were considered significant. For VOC meeting significance criteria, data are presented as the mean ± standard deviation of the normalized, transformed, and scaled abundance of each VOC.

Partial least squares discriminant analysis (PLS-DA) was used to determine whether the observed VOCs contributed to the classification of samples as originating from different groups. PLS-DA is a supervised approach that uses the abundance of each VOC to maximize the separation between the different groups in the first few dimensions (latent variables) [71, 72]. These latent variables are ranked by how well they explain the variance of the groups. To validate the models, the Q^2^ parameter was calculated as a measure of class prediction ability using 10-fold cross validation [73, 74]. The Q^2^ value was then compared to the distribution of Q^2^ values obtained from models of the same data using random permutations of group labels. This way, via 1000 permutation tests, statistical significance (*P*-value) of the given classification model was obtained [73, 74].

To determine which VOCs were most responsible for the classification, a variable importance in projection (VIP) score was calculated for each VOC [75, 76]. VOCs with higher VIP contribute more to the PLS-DA classification model. The VIP is calculated as a weighted sum of the squared correlations between the PLS-DA latent variables and the original variable [75, 76]. The weights correspond to the percentage of variation explained by the PLS-DA latent variable in the model. This study is the first application of robust statistics to maned wolf chemical communication and these methods can serve as an example for future semiochemical research in other species.

### VOC identification

The differentially expressed VOCs were identified by matching experimental spectra to that of known, authentic standards where available. When authentic standards were unavailable or cost-prohibitive to obtain, compounds were identified by first exporting deconvoluted spectra from the Automated Mass Spectral Deconvolution and Identification System (AMDIS, ver. 2.73) software into NIST MS Search (ver. 2.3) and searched against the Wiley Registry 11^th^ Edition/NIST 2017 combined mass spectral library (John Wiley and Sons, Inc., Hoboken, NJ; National Institute of Standards and Technology, Gaithersburg, MD). When library matches were ambiguous, likely VOC structures were drawn in ACD-ChemSketch (ver. 14.01) and exported to NIST MS Interpreter (ver. 3.4b) where mass spectral peaks and neutral losses were matched to fragments of the structure. When all major spectral peaks were accounted for, SMILES notations were created by ACD-ChemSketch and searched in NIH’s PubChem online database for naming. Compounds that could not be identified by this process were labeled as “unknown” with a given quantitative ion and retention time. In seven cases, compounds were identified as contaminants (column bleed or plasticizers) and were removed from further analysis.

## Results

### Abundant VOCs

A total of 113 peaks, each representing a VOC, were aligned across the 317 urine samples. Two VOC were found in 100% of the samples: 2,5-dimethyl pyrazine and 2-methyl-6-(1-propenyl)-pyrazine. For male maned wolf samples (n = 6 individuals, 141 samples), the most abundant VOCs were 2,5-dimethyl pyrazine, 2-methyl-6-(1-propenyl)-pyrazine, and 2-ethenyl-6-methyl-pyrazine. For female maned wolf samples (n = 6 individuals, 161 samples), the most abundant VOCs were 2,5-dimethyl pyrazine, 2-methyl-6-(1-propenyl)-pyrazine, and 3-ethyl 2,5-dimethyl pyrazine. The neutered male (n = 1 individual, n = 15 samples) showed much lower mean raw abundance overall. In this individual, VOCs with highest abundance were 2,5-dimethyl pyrazine, 4-heptanone, and 2-ethenyl-6-methyl-pyrazine (Table 2).

**Table 2:** VOC found in high abundance and with high frequency across maned wolf urine samples.

Compound	RT	CAS No.	Identification Method^b^	Raw Abundance		% Samples where Present
				Mean	SD	
**Males (n = 6 individuals, 141 samples)**						
2,5-dimethyl pyrazine	9.29	123-32-0	S	44104661	19238004	100.0
2-methyl-6-(1-propenyl)-pyrazine	13.57	18217-81-7	NIST17	32153093	19321932	100.0
2-ethenyl-6-methyl-pyrazine	12.47	13925-09-2	S	8608044	9157320	100.0
3-ethyl 2,5-dimethyl pyrazine	11.57	13360-65-1	NIST17	5844753	6781037	100.0
methyl prenyl sulfide	7.00	5897-45-0	NIST17 / MSI	5712228	13049045	100.0
geranyl nitrile	21.36	5146-66-7	NIST17	5514777	14339874	90.8
prenol	9.25	556-82-1	S	3453591	2669099	100.0
(Z)-2-penten-2-ol	4.39	61923-54-4	NIST17 / MSI	3445526	2208430	75.9
prenyl bromide	12.48	870-63-3	NIST17	2982101	9064296	99.3
2-isopropyl-5-methylpyrazine	10.74	13925-05-8	NIST17	1821186	1676289	87.9
**Females (n = 6 individuals, 161 samples)**						
2,5-dimethyl pyrazine	9.29	123-32-0	S	43198752	19896212	100.0
2-methyl-6-(1-propenyl)-pyrazine	13.57	18217-81-7	NIST17	15685846	14255934	100.0
3-ethyl 2,5-dimethyl pyrazine	11.57	13360-65-1	NIST17	2882259	2472631	96.9
4-heptanone	5.88	123-19-3	S	2545357	1949697	96.3
(Z)-2-penten-2-ol	4.39	61923-54-4	NIST17 / MSI	2406959	1620815	73.3
prenol	9.25	556-82-1	S	1769883	1673904	98.8
2-ethenyl-6-methyl-pyrazine	12.47	13925-09-2	S	1590649	2690336	95.7
2-isopropyl-5-methylpyrazine	10.74	13925-05-8	NIST17	1414256	1190236	91.9
methyl prenyl sulfide	7.00	5897-45-0	NIST17 / MSI	941837	1840391	98.8
trimethyl-pyrazine	10.99	14667-55-1	NIST17	815179	2095624	71.4
**Neutered Male (n = 1 individual, 15 samples)**						
2,5-dimethyl pyrazine	9.29	123-32-0	S	1808665	2061746	100.0
4-heptanone	5.88	123-19-3	S	540474	455294	100.0
2-ethenyl-6-methyl-pyrazine	12.47	13925-09-2	S	454826	533752	100.0
2-ethyl-1-hexanol	12.40	104-76-7	NIST17 / MSI	310368	393021	93.3
acetophenone	15.97	98-86-2	S	288733	723863	100.0
2-isopropyl-5-methylpyrazine	10.74	13925-05-8	NIST17	274991	448499	86.7
N-acetylpyrrole	13.68	609-41-6	NIST17 / MSI	164406	84832	100.0
4-butoxy-2-butanone	8.95	30536-44-8	NIST17 / MSI	163595	135777	86.7
benzaldehyde	13.27	100-52-7	S	161798	206243	100.0
3-ethylcyclopentanone	9.50	10264-55-8	S	122202	89567	93.3

^a^Identification method

S = Experimental spectrum matched to authentic standard.

N17 = Experimental spectrum matched to NIST17 spectral library.

MSI = Experimental spectrum ion fragments matched to structure fragments in NIST MS Interpreter.

### Differences between male and female urinary VOCs

**Fig 1:**
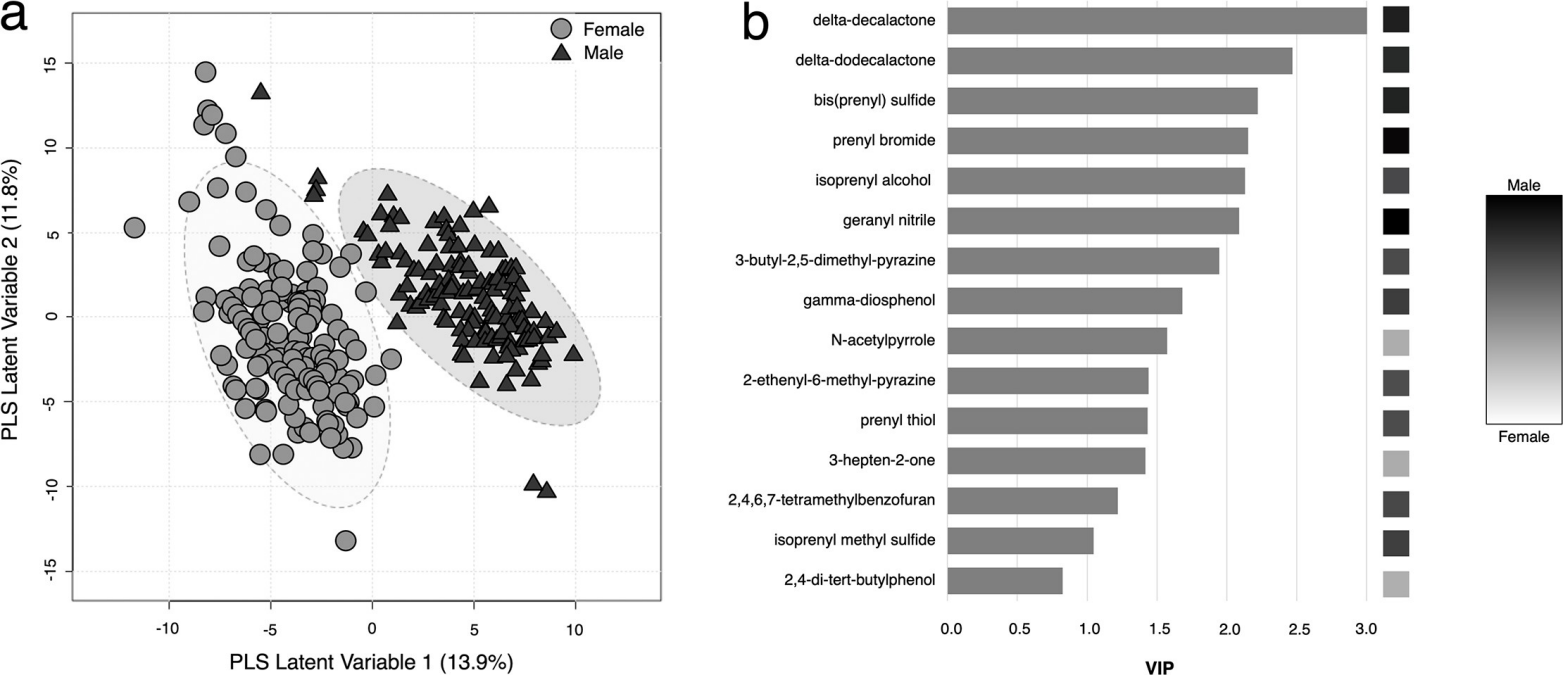
Classification of male and female urine samples based on abundance of 76 VOC. (a) Scores on first 2 latent variables from partial least squares-discriminant analysis shown for female samples (n = 161) in gray circles and male samples (n = 141) in black triangles. Variance explained by each latent variable is shown in parentheses. (b) Variable influence on projection (VIP) scores on partial least squares latent variable 1 shown for 15 VOCs with statistically significant differential expression. Boxes on right indicate the log_2_ fold change in abundance of each VOC in male samples (n = 141) compared to female samples (n = 161).

Following peak picking and grouping procedures, 76 peaks, each representing a VOC, were analyzed for differential expression between males (n = 6 individuals, 141 samples) and females (n = 6 individuals, 161 samples). Fifteen urinary VOCs were found to differ significantly (*Adj*. *P* < 0.001 and |log_2_ fold change| > 2.0) between males and females (Table 3). Of those, twelve VOCs were higher in abundance in males than females and the other three showed higher abundance in females than males. In the PLS-DA, the 76 VOCs were reduced to five PLS latent variables with good discrimination ability between males and females (Q^2^ = 0.90, permuted *P* < 0.001) (Fig 1A). The first latent variable accounted for 13.9% of the explained variance and related to the sex differences. Compounds that were higher in abundance in female urine had negative loadings, while compounds that were higher in male urine had positive loadings. Compounds most influential to the classification were: delta-decalactone, delta-dodecalactone, bis(prenyl) sulfide, prenyl bromide, and isoprenyl alcohol (Fig 1B).

**Table 3:** VOCs that differed significantly between male and female maned wolf urine samples.

Compound^a^	RT	CAS No.	Identification Method^b^	Normalized Abundance mean ± SD		Log_2_ Fold Change	*Adj*. *P*	Variable Importance on Projection
				Male N = 6 wolves N = 141 samples	Female N = 6 wolves N = 161 samples			
delta-decalactone	28.61	705-86-2	NIST17 / MSI	1.66 ± 1.27	-1.45 ± 1.15	4.60	4.33E-64	3.00
delta-dodecalactone	29.25	713-95-1	NIST17 / MSI	1.37 ± 1.39	-1.20 ± 0.62	4.01	9.48E-60	2.47
bis(prenyl) sulfide	14.47	Pubchem# 11095069	MSI	1.23 ± 1.33	-1.08 ± 1.49	4.44	1.00E-33	2.22
prenyl bromide	12.67	870-63-3	NIST17 / MSI	1.19 ± 1.30	-1.04 ± 1.35	5.74	1.55E-35	2.15
isoprenyl alcohol	3.86	115-18-4	S	1.16 ± 1.79	-1.01 ± 1.64	2.60	4.64E-31	2.13
geranyl nitrile	21.35	5146-66-7	NIST17	1.18 ± 1.58	-1.03 ± 1.30	6.09	7.39E-23	2.09
3-butyl-2,5-dimethyl-pyrazine	15.01	40790-29-2	NIST17	1.08 ± 1.56	-0.94 ± 1.57	2.46	1.94E-23	1.95
gamma-diosphenol	13.54	54783-36-7	NIST17 / MSI	0.93 ± 1.44	-0.82 ± 0.62	3.04	2.62E-33	1.68
N-acetylpyrrole	13.68	609-41-6	NIST17 / MSI	-0.87 ± 1.63	0.76 ± 1.53	-2.34	2.04E-16	1.57
2-ethenyl-6-methyl-pyrazine	12.47	13925-09-2	S	0.80 ± 1.55	-0.70 ± 1.56	2.26	1.65E-14	1.44
prenylthiol	27.36	5287-45-6	NIST17	0.79 ± 1.94	-0.69 ± 1.76	2.39	7.67E-11	1.43
3-hepten-2-one	8.91	1119-44-4	S	-0.78 ± 1.06	0.69 ± 1.66	-2.30	1.32E-16	1.42
2,4,6,7-tetramethylbenzofuran	21.80	Pubchem# 101630179	NIST17	0.68 ± 1.60	-0.59 ± 0.77	2.56	2.04E-16	1.22
isoprenyl methyl sulfide	19.18	5952-75-0	NIST17 / MSI	0.58 ± 1.87	-0.51 ± 1.69	3.01	7.45E-07	1.05
2,4-di-tert-butylphenol	29.55	96-76-4	NIST17	-0.46 ± 1.18	0.40 ± 1.60	-2.32	1.05E-06	0.82

^a^Compounds listed are those that met significance criteria of *Adj*. *P* < 0.001 and |log_2_ fold change| > 2.0.

^b^Identification method

S = Experimental spectrum matched to authentic standard.

N17 = Experimental spectrum matched to NIST17 spectral library.

MSI = Experimental spectrum ion fragments matched to structure fragments in NIST MS Interpreter.

### Differences between intact and neutered male urinary VOCs

**Fig 2:**
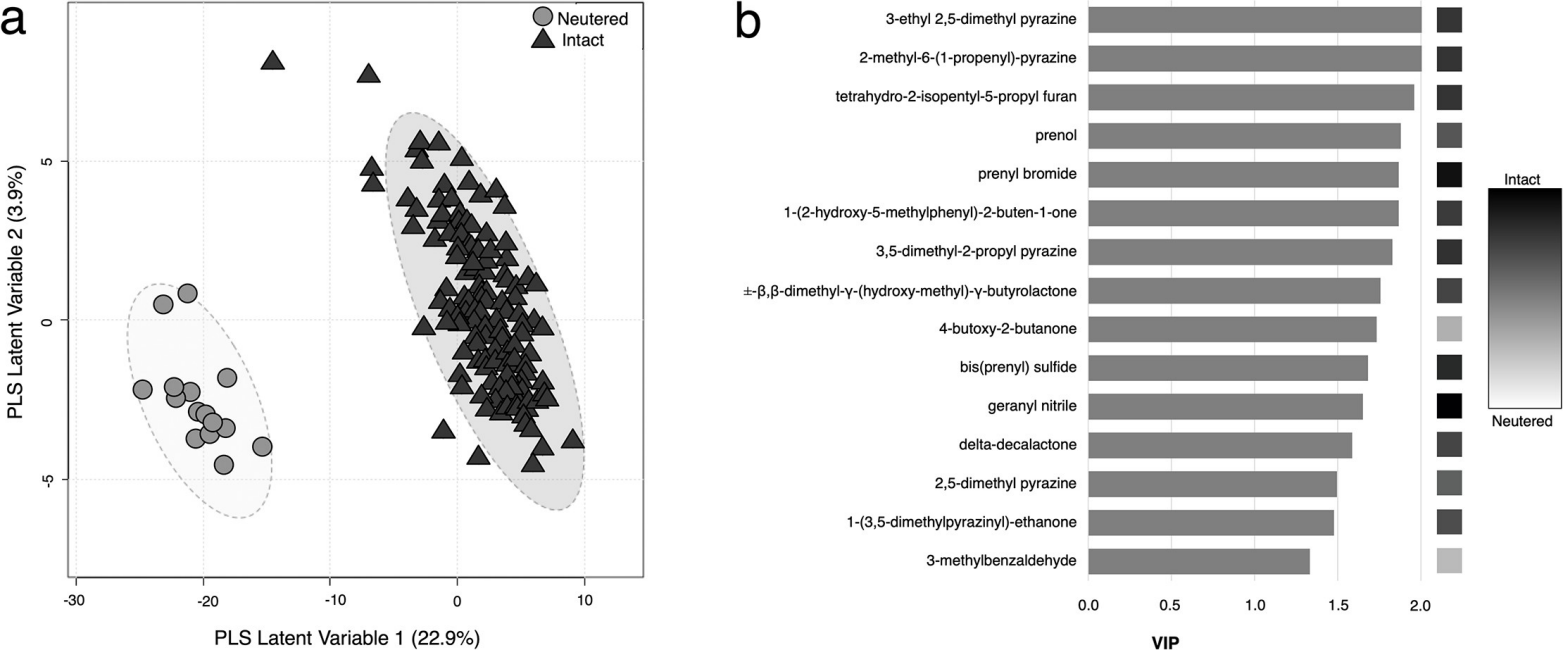
VOCs with highest variable importance in classifying intact male and neutered male urine samples. (a) Scores on first 2 latent variables from partial least squares-discriminant analysis shown for neutered male samples (n = 15) in gray circles and intact male samples (n = 141) in black triangles. Variance explained by each latent variable is shown in parentheses. (b) Variable influence on projection (VIP) scores on partial least squares latent variable 1 shown for 15 VOCs with statistically significant differential expression and highest VIP. Boxes on right indicate the log_2_ fold change in abundance of each VOC in intact male samples (n = 141) compared to a neutered male’s samples (n = 15).

Following peak picking and grouping procedures, 98 peaks each representing a VOC were analyzed for differential expression between intact males (n = 6 individuals, 141 samples) and a neutered male (n = 1 individual, 15 samples). Out of those 98 peaks, 62 urinary VOCs were found to differ significantly (*Adj*. *P* < 0.001 and |log_2_ fold change| > 2.0) between intact males and the neutered male (Table 4). Of those, 26 VOCs were higher in abundance in intact males than neutered and the other 36 showed higher abundance in the neutered male samples than in those from intact males. The 98 VOCs were reduced to five PLS latent variables with good discrimination ability between intact males and the neutered male (Q^2^ = 0.93, permuted *P* < 0.001) (Fig 2A). The first latent variable, accounting for 22.9% of the explained variance, related to the difference in intact males compared to the neutered individual. Compounds that were higher in abundance in urine samples from the neutered male had negative loadings, while compounds that were higher in abundance in intact males’ urine had positive loadings. Compounds with the highest contribution to the classification were: 3-ethyl 2,5-dimethyl pyrazine, 2-methyl-6-(1-propenyl) pyrazine, tetrahydro-2-isopentyl-5-propyl furan, prenol, and prenyl bromide (Fig 2B). Of the top ten compounds with the highest contribution to the classification, nine were significantly more abundant in the urine of intact males.

**Table 4:** VOCs that differed significantly between intact and neutered male maned wolf urine samples.

Compound^a^	RT	CAS No.	Identification Method^b^	Normalized Abundance mean ± SD		Log_2_ Fold Change	*Adj*. *P*	Variable Importance on Projection
				Intact Male N = 6 wolves N = 141 samples	Neutered Male N = 1 wolf N = 15 samples			
3-ethyl 2,5-dimethyl pyrazine	11.57	13360-65-1	NIST17	0.46 ± 0.94	-4.31 ± 1.80	6.84	3.46E-35	2.09
2-methyl-6-(1-propenyl)-pyrazine	13.57	18217-81-7	NIST17	0.45 ± 0.77	-4.20 ± 1.05	6.96	3.90E-46	2.04
tetrahydro-2-isopentyl-5-propyl furan	23.16	33933-71-0	NIST17	0.43 ± 0.91	-4.04 ± 0.94	6.96	3.59E-38	1.96
prenol	9.25	556-82-1	S	0.41 ± 0.71	-3.87 ± 2.21	3.85	7.48E-35	1.88
prenyl bromide	12.48	870-63-3	NIST17	0.41 ± 1.27	-3.85 ± 0.94	10.14	1.79E-24	1.87
isoprenyl methyl sulfide	19.09	5952-75-0	NIST17	0.41 ± 0.95	-3.85 ± 0.96	6.27	2.05E-34	1.87
3,5-dimethyl-2-propyl pyrazine	12.97	32350-16-6	NIST17	0.40 ± 1.19	-3.77 ± 0.99	7.15	8.50E-26	1.83
±-β,β-dimethyl-γ-(hydroxy-methyl)-γ-butyrolactone	19.36	52398-48-8	NIST17	0.39 ± 0.83	-3.62 ± 1.04	5.50	1.79E-36	1.76
4-butoxy-2-butanone	8.95	30536-44-8	NIST17 / MSI	-0.38 ± 1.00	3.57 ± 1.52	-4.65	1.92E-27	1.73
bis(prenyl) sulfide	14.48	Pubchem# 11095069	MSI	0.37 ± 1.38	-3.47 ± 0.86	7.87	5.45E-19	1.68
geranyl nitrile	21.36	5146-66-7	NIST17	0.36 ± 1.78	-3.40 ± 0.74	11.52	7.07E-13	1.65
delta-decalactone	28.61	705-86-2	NIST17 / MSI	0.35 ± 1.42	-3.27 ± 1.26	5.33	2.59E-16	1.59
2,5-dimethyl pyrazine	9.29	123-32-0	S	0.33 ± 0.70	-3.08 ± 1.85	2.81	3.02E-29	1.49
1-(3,5-dimethylpyrazinyl)-ethanone	16.44	54300-08-2	S	0.32 ± 1.09	-3.04 ± 1.06	4.64	3.02E-21	1.48
3-methylbenzaldehyde	15.91	620-23-5	NIST17	-0.29 ± 0.79	2.74 ± 2.26	-5.42	3.01E-20	1.33
N-acetylpyrrole	13.68	609-41-6	NIST17 / MSI	-0.29 ± 1.62	2.72 ± 0.85	-3.68	1.72E-10	1.32
3-acetyl-2,4-dimethylfuran	14.38	NA	NIST17	-0.29 ± 1.18	2.71 ± 0.97	-3.47	2.36E-16	1.32
prenol	9.21	5287-45-6	NIST17 / MSI	-0.29 ± 1.40	2.70 ± 1.39	-3.62	2.82E-12	1.31
2,5-dimethyl-3-(3-methylbutyl)-pyrazine	16.05	18433-98-2	NIST17	0.29 ± 1.62	-2.69 ± 0.84	5.88	2.01E-10	1.31
4-methyl-2-heptanone	7.36	6137-06-0	NIST17	-0.29 ± 0.94	2.69 ± 2.32	-4.71	8.52E-17	1.31
2-ethyltetrahydrofuran	16.76	1003-30-1	NIST17 / MSI	-0.29 ± 0.94	2.69 ± 1.55	-3.67	6.11E-20	1.31
nonanal	10.64	124-19-6	S	-0.28 ± 1.51	2.63 ± 1.20	-3.68	9.04E-11	1.27
methyl ester nonanoic acid	12.56	1731-84-6	S	-0.27 ± 1.24	2.58 ± 1.61	-3.60	3.65E-13	1.25
3-butyl-2,5-dimethyl-pyrazine	15.01	40790-29-2	NIST17	0.26 ± 1.65	-2.47 ± 0.85	5.43	7.22E-09	1.20
tetrahydro-2,5-dimethyl-2H-pyranmethanol	10.93	54004-46-5	NIST17	-0.25 ± 1.09	2.32 ± 1.73	-4.07	4.62E-13	1.13
N,N-dibutyl-formamide	18.58	761-65-9	S	-0.24 ± 1.03	2.25 ± 1.68	-3.70	2.24E-13	1.09
diphenylamine	34.76	122-39-4	S	-0.24 ± 0.94	2.22 ± 1.48	-3.59	3.57E-15	1.08
prenylthiol	27.37	5287-45-6	NIST17	0.24 ± 1.94	-2.21 ± 0.80	7.11	7.14E-06	1.07
2-pentyl furan	7.70	3777-69-3	NIST17	-0.24 ± 0.81	2.21 ± 1.16	-2.70	3.13E-19	1.07
unknown 176@30.01	30.01			-0.23 ± 0.93	2.21 ± 0.89	-2.53	6.97E-17	1.07
2,4-di-tert-butylphenol	29.56	96-76-4	NIST17	-0.23 ± 1.23	2.18 ± 1.46	-3.10	1.72E-10	1.06
isoprenyl alcohol	3.86	115-18-4	S	0.23 ± 1.66	-2.14 ± 0.87	4.77	4.73E-07	1.04
isophorone	10.85	78-59-1	NIST17	-0.22 ± 1.16	2.06 ± 1.67	-3.12	4.00E-10	1.00
3-hepten-2-one	8.93	1119-44-4	S	-0.22 ± 1.15	2.05 ± 2.06	-3.76	1.60E-09	1.00
5-methyl-5-(1-methylethyl)-3-heptyne-2,6-dione	11.29	63922-44-1	NIST17	-0.22 ± 1.00	2.05 ± 1.51	-2.73	1.61E-12	0.99
2-methyl, 3-hydroxy-2,4,4-trimethylpentyl ester propanoic acid	20.67	74367-34-3	NIST17	-0.22 ± 1.73	2.04 ± 1.28	-1.98	4.82E-06	0.99
delta-dodecalactone	29.25	713-95-1	NIST17 / MSI	0.21 ± 1.48	-1.99 ± 0.96	3.64	1.99E-07	0.96
trimethyl-pyrazine	10.99	14667-55-1	NIST17	0.20 ± 2.00	-1.91 ± 0.75	6.14	1.32E-04	0.93
methyl 1-methyl-2-butenyl sulfide	19.18	89534-73-6	NIST17	0.20 ± 1.85	-1.90 ± 0.80	6.71	4.51E-05	0.92
tert-butyl pivalate	14.94	16474-43-4	NIST17	-0.20 ± 1.10	1.88 ± 1.81	-3.08	3.02E-09	0.91
1,2-dibutyl-hydrazine	12.49	1744-71-4	NIST17	-0.20 ± 0.93	1.87 ± 1.74	-3.11	2.53E-11	0.91
1-octanol	13.82	111-87-5	S	-0.20 ± 1.31	1.87 ± 1.42	-2.51	1.15E-07	0.91
(Z)-2-penten-2-ol	4.39	61923-54-4	NIST17 / MSI	0.20 ± 2.10	-1.87 ± 1.87	2.10	5.51E-04	0.91
2,6-ditert-butyl-4-hydroxy-4-methylcyclohexa-2,5-dien-1-one	25.51	NA	NIST17	-0.18 ± 0.97	1.71 ± 1.36	-2.28	3.79E-10	0.83
diphenyl ether	23.68	101-84-8	NIST17 / MSI	-0.18 ± 1.04	1.71 ± 1.19	-1.83	1.86E-09	0.83
1-(4-aminophenyl)ethanone oxime	7.20	38063-81-9	NIST17	-0.18 ± 1.14	1.70 ± 1.68	-2.36	1.15E-07	0.82
2-isopropyl-5-methylpyrazine	10.74	13925-05-8	NIST17	0.18 ± 1.10	-1.70 ± 1.78	1.55	7.04E-08	0.82
benzaldehyde	13.27	100-52-7	S	-0.18 ± 1.70	1.70 ± 1.12	-1.89	9.32E-05	0.82
3-octen-2-one	10.90	1669-44-9	S	-0.17 ± 1.37	1.64 ± 1.65	-2.04	8.13E-06	0.80
tri-sec-butyl ester orthoformic acid	6.02	16754-48-6	NIST17	-0.17 ± 1.62	1.62 ± 1.36	-2.02	1.12E-04	0.78
2-tert-butyl-3-(3,3-dimethylbutyl)oxirane	25.09	NA	MSI	0.17 ± 1.49	-1.61 ± 0.98	2.95	2.35E-05	0.78
1-octen-3-ol	11.59	3391-86-4	S	-0.17 ± 1.64	1.59 ± 1.00	-1.86	1.23E-04	0.77
3-ethylcyclopentanone	9.50	10264-55-8	S	-0.17 ± 1.42	1.58 ± 0.95	-2.13	1.28E-05	0.77
methyl 2-phenylacetate	18.34	101-41-7	NIST17 / MSI	-0.17 ± 1.25	1.57 ± 2.41	-4.35	1.94E-05	0.76
1-hexanol	9.74	111-27-3	S	-0.17 ± 1.28	1.55 ± 1.52	-2.04	6.24E-06	0.75
2-ethenyl-6-methyl-pyrazine	12.50	13925-09-2	S	0.16 ± 1.63	-1.51 ± 0.79	3.55	2.13E-04	0.73
3-tert-butyl-2-pyrazolin-5-one	18.30	29211-68-5	S	-0.15 ± 1.32	1.43 ± 1.23	-2.21	3.42E-05	0.69
2-acetyl-3,4,6-trimethylpyrazine	18.50	125186-38-1	NIST17 / MSI	0.15 ± 1.31	-1.40 ± 1.10	2.05	3.53E-05	0.68
endo-borneol	17.00	507-70-0	NIST17 / MSI	-0.15 ± 1.26	1.40 ± 1.55	-1.72	3.28E-05	0.68
(E)-2,3-dimethyl-5-(1-propenyl) pyrazine	15.20	80033-12-1	NIST17 / MSI	0.15 ± 1.52	-1.37 ± 0.98	2.89	3.74E-04	0.66
trans-β-ionone	22.20	79-77-6	NIST17 / MSI	-0.13 ± 1.30	1.26 ± 1.19	-1.54	1.70E-04	0.61
2,6-dimethyl-2,6-octadiene	17.90	2792-39-4	NIST17 / MSI	-0.13 ± 1.36	1.24 ± 1.56	-1.58	5.20E-04	0.60

^a^Compounds listed are those that met significance criteria of *Adj*. *P* < 0.001 and |log_2_ fold change| > 2.0.

^b^Identification method

S = Experimental spectrum matched to authentic standard.

N17 = Experimental spectrum matched to NIST17 spectral library.

MSI = Experimental spectrum ion fragments matched to structure fragments in NIST MS Interpreter.

### Differences between paired and unpaired individuals

VOCs from males paired with a breeding mate (SB#2660 and SB#2844) (n = 44 samples) were compared to those from males housed without a breeding mate (SB#3206, SB#3192, SB#3195, SB#2810) (n = 97 samples). Following peak picking and grouping procedures, 73 peaks each representing a VOC were analyzed for differential expression. Out of those 73 peaks, four urinary VOCs differed (*Adj*. *P* < 0.001 and |log_2_ fold change| > 2.0) between paired and unpaired males: delta-dodecalactone, 1-(5-methyl-2-pyrazinyl)-1-ethanone, methyl ester benzoic acid, and bis(prenyl) sulfide (S1 Table). Of those, two VOCs were higher in abundance in paired males than unpaired males (methyl ester benzoic acid and bis(prenyl) sulfide) and the other two showed higher abundance in the unpaired male samples than in those from paired males (delta-dodecalactone and 1-(5-methyl-2-pyrazinyl)-1-ethanone). The 73 VOCs were reduced to five PLS latent variables that had lower discrimination ability than the other analyses (Q^2^ = 0.57, permuted *P* < 0.001), demonstrating that these two groups were not as easily discriminated as males and females or intact and neutered males.

Paired females (SB#2945 and SB#3184) (n = 41 samples) were compared to unpaired females (SB#3231, SB#3232, SB#2845) (n = 96 samples). Following peak picking and grouping procedures, 78 peaks each representing a VOC were analyzed for differential expression. Out of those 78 peaks, four urinary VOCs differed between the two groups (*Adj*. *P* < 0.001 and |log_2_ fold change| >2.0): 1-hexanol, 1-octen-3-ol, benzyl methyl ketone, and 3-ethyl-2,5-dimethyl-pyrazine (S2 Table). Of those, all four VOCs were higher in abundance in unpaired females than paired females. The 78 VOCs were reduced to five PLS latent variables (Q^2^ = 0.76, permuted *P* < 0.001).

## Discussion

This study provides the most comprehensive analysis of maned wolf urinary VOCs to date and is the first study to investigate differential expression of maned wolf urinary VOCs by sex, male reproductive status (intact or neutered), and pairing status. Through the use of chemical structure matching software, several hemiterpenoid compounds originating from the mevalonate pathway were accurately identified. Two VOCs were found in all 317 samples, 2,5-dimethyl pyrazine and 2-methyl-6-(1-propenyl)-pyrazine. Fifteen VOCs differed significantly between males and females while 62 VOCs differed between intact males and a neutered male. Several VOCs established as important here have been implicated in reproductive communication in other mammals. Based on their differential expression, several putative semiochemicals were identified as good candidates for behavioral bioassays, in particular delta-decalactone and delta-dodecalactone.

There is robust evidence that the urinary VOCs of maned wolves differ significantly based on sex, with the male producing higher abundances of many VOCs. Of the 15 VOCs that differed by sex, 12 were more abundant in males than females. This supports evidence in rodents [77] and primates [78, 79] that males typically produce a greater repertoire and intensity of odors, possibly playing a role in territory defense. Although there are a few species that do not conform to this trend (for example: cotton-top tamarin, *Sanguinus oedipus*) [79], this effect is likely due to male biased sexual dimorphism in the number and size of glands [78, 79].

The VOCs that were the strongest indicators of sex were delta-decalactone, delta-dodecalactone, bis(prenyl) sulfide, prenyl bromide, and isoprenyl alcohol. These five VOCs were all higher in relative abundance in males and delta-decalactone and bis(prenyl) sulfide differed by pairing status. Delta-decalactone and delta-dodecalactone have a sweet, coconut odor [80, 81] and are primary odor compounds in the warning secretion on porcupine (*Erethizon dorsatum*) quills [82], and in the marking fluid of the male bengal tiger (*Panthera tigris*) [83]. Literature references to bis(prenyl) sulfide and prenyl bromide as scent signaling compounds in other species were not found, however bis(prenyl) sulfide may be the result of prenylation of proteins containing terminal sulfides [84] and prenyl bromide forms methyl prenyl sulfide [85], a compound found in maned wolf urine samples in Goodwin et al. [47] and in the present analysis. Isoprenyl alcohol is a constituent of the interdigital gland secretion of both male and female red hartebeest (*Alcelaphus buselaphus caama*) [86] and is a pheromone in several hundred species of insect [87, 88]. Future studies examining production of these compounds correlated to hormone excretion over a year would enable comparisons between breeding and non-breeding season, lending additional support to their identities as semiochemicals in this species.

Importantly, the identifications of the delta-lactones and prenyl bromide were based on spectral library searches and verified by matching the ion peaks in the experimental spectrum with structural fragments using NIST MS Interpreter. The identification of bis(prenyl) sulfide, a hemiterpenoid thioether, was based on a matching ion fragment peaks with those in prenyl thiol, a hemiterpenoid thiol compound previously identified in maned wolf urine [47]. The prenyl thiol spectrum has nearly identical fragments as those found in the experimental spectrum with the exception of an additional hydrogen. The experimental spectrum was then matched to the structure of (bis)prenyl sulfide and all ion peaks were accounted for by structural fragments.

Striking differences were noted between samples from intact males and the neutered male. Of the five VOCs that contributed the most to the classification as intact or neutered, all were more abundant in urine samples from intact males compared to those from the neutered male. 3-Ethyl 2,5-dimethyl pyrazine, one of the main VOCs in maned wolf urine [47], is a trail pheromone and attractant for several Hymenoptera sp. [89]. 2-Methyl-6-(1-propenyl) pyrazine, also a main VOC of maned wolf urine [47, 58, 59], was around 7-fold less abundant in the samples from the neutered male compared to those from intact males. Tetrahydro-2-isopentyl-5-propyl furan is a VOC emitted from fresh cherries (*Prunus avium lapins*) [90]. Although no reference was found for tetrahydro-2-isopentyl-5-propyl furan in mammalian semiochemical literature, furans are very common as mammalian scent constituents. For example, 2-methyl furan is a urinary VOC for the gray wolf [56], three furans are urinary VOCs in the African wild dog [39], and several furans are more abundant in intact male mice compared to neutered males [91]. Prenol was previously identified in maned wolf urine [47, 59] and is also found in the pre-orbital secretion of the blue duiker (*Cephalophus monticola*) [92] and in anal gland secretions of the mink (*Mustela vison*) [93].

Even to the human nose, urine from the neutered male did not smell strongly at all while the urine samples from intact males were quite pungent. Out of the ten most significantly different VOCs, nine were more abundant in intact males as compared to samples from the neutered male, suggesting that these compounds may be dependent on testosterone. Hormone-mediated odor production is well studied in other species. In the gray wolf, production of urinary VOCs usually associated with intact males was induced by testosterone treatment of castrated males [57]. In primates, contraceptive treatment of females alters her genital odor profile, making her smell less distinctive [94]. In small rodents, neutered males lose many behaviors that are associated with urinary pheromones. The ability to attract females (in mice, *Mus musculus*) [95, 96], investigate the female’s ano-genital region and copulate (in Syrian hamsters, *Mesocricetus auratus*) [97], accelerate puberty in females (in prairie voles, *Microtus ochrogaster*) [98, 99], and prevent implantation (in mice) [100] are pheromonal effects. When neutered males are hormonally treated, these behaviors are reinstated [99, 100]. Treatment of our neutered male with testosterone was not possible during this study, but would certainly lead to important discoveries about the testosterone dependence of these VOCs.

Thirteen VOCs were found to be significantly different in both analyses by sex and reproductive status. Of those, ten were higher in abundance in males than females and more abundant in intact males than the neutered male: delta-decalactone, delta-dodecalactone, bis(prenyl) sulfide, prenyl bromide, isoprenyl alcohol, geranyl nitrile, 3-butyl-2,5-dimethyl-pyrazine, 2-ethenyl-6-methyl-pyrazine, prenyl thiol, and isoprenyl methyl sulfide. It is reasonable to hypothesize that these VOCs indicate “maleness” or the presence of testosterone. Behavioral bioassays with this suite of VOCs would help to further elucidate their biological roles in the maned wolf. Females in estrus should display more behavioral interest to VOCs modulated by testosterone than to VOCs that are present in maned wolf urine but are not related to reproduction.

This study provides an excellent foundation for selecting VOCs to bioassay that may play a role in ovulation induction in the maned wolf. In particular, delta-decalactone and delta-dodecalactone deserve closer attention in bioassay as they were each four-fold higher in normalized abundance in males than females, higher in intact males than the neutered male, and are known signaling compounds in other mammals [82, 83]. Several hemiterpenoid compounds are worthy of further investigation. Bis(prenyl) sulfide had 4-fold higher normalized abundance in males compared to females and an 8-fold increase in normalized abundance in intact males compared to the neutered male and was 2-fold higher in paired males over unpaired males. Prenyl bromide was 6-fold higher in males compared to females and 10-fold higher in intact males compared to the neutered individual. Isoprenyl alcohol was 2 times more prevalent in males compared to females and 5-fold higher in intact males over the neutered male, and finally geranyl nitrile was 6-fold higher in males compared to females and 11-fold more abundant in intact males over the neutered male. These prenyl compounds are synthesized via the mevalonate pathway which begins with isopentenyl diphosphate and dimethylallyl diphosphate, the building blocks of cholesterol and steroid hormones [101]. Because of their link to reproductive hormones, the identification of these compounds as differentially expressed in maned wolves according to sex and reproductive status is especially intriguing and deserving of bioassay. A future study investigating the correlation of urinary VOC changes to hormonal and behavioral changes across the year would further elucidate the role of these compounds.

Like the three previous investigations of maned wolf urinary VOCs, the present study found pyrazine compounds were some of the most prevalent [47, 58, 59]. Goodwin et al. reports high prevalence of 2,5-dimethyl pyrazine, 2-methyl-6-(1-propenyl)-pyrazine, 2-ethenyl-6-methyl pyrazine, and 3-ethyl 2,5-dimethyl pyrazine [47], all of which were found in over 70% of both male and female samples in the present study, though notably, only 2,5-dimethyl pyrazine and 2-ethenyl-6-methyl pyrazine were prevalent (over 70% of samples and top 10 by mean raw abundance) in the neutered male samples. All of these pyrazines were also identified in all 103 maned wolf urine samples in Kester et al. [59]. Childs-Sanford identified 2,5-dimethyl pyrazine and 2-methyl-6-(1-propenyl)-pyrazine as chemical constituents found in high concentrations across seven urine samples [58]. Pyrazines are common semiochemicals among insects [102] and are also signaling compounds in several taxa of mammals, including as components of gray wolf urine causing freezing behavior in mice [103–105]. Findings here support previous hypotheses that pyrazines are responsible for the characteristic pungent odor of maned wolf urine [47, 58].

Terpenoid compounds are widely found throughout nature as scent compounds [106]. Additionally, they are prevalent as urinary VOCs in the maned wolf [47, 59]. In the present study, methyl prenyl sulfide and prenol were prevalent in both male and female samples. Notably, neither of these were identified as prevalent in samples from the neutered male. Methyl prenyl sulfide has a foul smell and aside from its prevalence in maned wolf urinary VOCs, is also a signaling compound in insect species [85]. Terpenoid compounds were very important to the classification of samples by sex, with bis(prenyl) sulfide, prenyl bromide, isoprenyl alcohol, geranyl nitrile, prenyl thiol, and isoprenyl methyl sulfide all significantly more abundant in males than in females. Prenol, prenyl bromide, (bis)prenyl sulfide, and geranyl nitrile were important to the classification of samples by male reproductive status; all four VOCs were more abundant in samples from intact males compared to those from the neutered male. Additionally, (bis)prenyl sulfide was more abundant in urine from paired males when compared to samples from unpaired males. The origin of terpenoid compounds is through the mevalonate pathway’s products prenyl pyrophosphate and isopentenyl pyrophosphate [107]. Prenyl pyrophosphate forms hemiterpenoid prenyl compounds such as prenol, prenyl bromide, methyl prenyl sulfide and bis(prenyl) sulfide. Prenyl pyrophosphate and isopentenyl phosphate give rise to the monoterpene geranyl compound geranyl nitrile [107]. The mevalonate pathway plays a critical role in the production of hormones and pheromones in insects [107], so the identification of so many terpenoid VOCs as differentially expressed by sex, reproductive status, and pairing status is intriguing.

## Conclusion

Despite conservation efforts in the wild, preservation of a species often requires maintaining healthy, *ex situ* populations to supplement declining *in situ* populations, to educate the public about the species, and to research the basic biology of the species in order to improve conservation efforts [6, 108]. As such, investigating species-specific aspects of wildlife reproduction, especially focusing on defining novel and unique reproductive mechanisms, is the highest priority of wildlife research today [109, 110].

These results add valuable information to the growing body of knowledge of mammalian semiochemistry and reproductive mechanisms. The maned wolf is only the fifth species (out of 36) within Canidae, and the only solitary canid species, to be investigated for urinary VOCs. Few of the existing canid studies have attempted to analyze differential expression by sex (excepting [39, 40, 56, 57]), and none have done so in the solitary maned wolf that relies on semiochemicals-mediated induced ovulation for reproductive success. New structural matching software allowed the identification of several prenyl compounds as prevalent or as differentially expressed according to sex or reproductive status and confirmed the identity of methyl prenyl sulfide in maned wolf urine. This work lays an important foundation for semiochemical discovery in this species and establishes a robust, open-source data analysis pipeline that can be widely adopted to improve differential analyses in other species to answer biologically relevant questions.

## Supporting information

S1 Table VOCs that differed significantly between paired and unpaired male maned wolf urine samples. (DOCX)

S2 Table VOCs that differed significantly between paired and unpaired female maned wolf urine samples. (DOCX)
