# Supplementary material for: Differential expression of urinary volatile organic compounds by sex, male reproductive status, and pairing status in the maned wolf (Chrysocyon brachyurus)
Source: PLoS One. 2021 Aug 20;16(8):e0256388. doi: 10.1371/journal.pone.0256388 (PMC8378691; doi:10.1371/journal.pone.0256388)
Supplement: S1 Table — (DOCX) [file pone.0256388.s001.docx]

**S1 Table. VOCs that differed significantly between paired and unpaired male maned wolf urine samples.**

| **Compound^a^** | **RT** | **CAS No.** | **Identification Method^b^** | **Normalized Abundance mean ± SD** | | **Log_2_ Fold Change** | ***Adj. P*** | **Variable Importance on Projection** |
| --- | --- | --- | --- | --- | --- | --- | --- | --- |
|  |  |  |  | **Paired Male samples (N = 44)** | **Unpaired Male samples (N = 97)** |  |  |  |
| delta-dodecalactone | 29.25 | 713-95-1 | NIST17 / MSI | -1.26 ± 1.47 | 0.57 ± 1.11 | -2.04 | 1.33E-11 | 2.35 |
| 1-(5-methyl-2-pyrazinyl)-1-ethanone | 16.66 | 22047-27-4 | NIST17 | -0.96 ± 1.22 | 0.44 ± 1.71 | -2.34 | 1.86E-05 | 1.79 |
| methyl ester benzoic acid | 15.35 | 93-58-3 | S | 0.75 ± 1.61 | -0.34 ± 1.51 | 2.13 | 5.74E-04 | 1.40 |
| bis(prenyl) sulfide | 14.48 | Pubchem# 11095069 | NIST17 / MSI | 0.69 ± 1.85 | -0.31 ± 1.28 | 2.41 | 9.86E-04 | 1.28 |

^a^Compounds listed are those that met significance criteria of *Adj. P* < 0.001 and |log_2_ fold change| > 2.0

^b^Identification method:

S = Experimental spectrum matched to authentic standard

N17 = Experimental spectrum matched to NIST17 spectral library

MSI = Experimental spectrum and presumed structure matched to compound ID in MS Interpreter
