# Supplementary material for: Differential expression of urinary volatile organic compounds by sex, male reproductive status, and pairing status in the maned wolf (Chrysocyon brachyurus)
Source: PLoS One. 2021 Aug 20;16(8):e0256388. doi: 10.1371/journal.pone.0256388 (PMC8378691; doi:10.1371/journal.pone.0256388)
Supplement: S2 Table — (DOCX) [file pone.0256388.s002.docx]

**S2 Table. VOCs that differed significantly between paired and unpaired female maned wolf urine samples.**

| **Compound^a^** | **RT** | **CAS No.** | **Identification Method^b^** | **Normalized Abundance mean ± SD** | | **Log_2_ Fold Change** | ***Adj. P*** | **Variable Importance on Projection** |
| --- | --- | --- | --- | --- | --- | --- | --- | --- |
|  |  |  |  | **Paired Female samples (N = 41)** | **Unpaired Female samples (N = 96)** |  |  |  |
| 1-hexanol | 9.73 | 111-27-3 | S | -1.15 ± 0.68 | 0.49 ± 1.53 | -3.30 | 7.22E-08 | 2.38 |
| 1-octen-3-ol | 11.59 | 3391-86-4 | S | -1.13 ± 1.34 | 0.48 ± 1.58 | -2.72 | 1.42E-06 | 2.34 |
| benzyl methyl ketone | 17.64 | 103-79-7 | NIST17 | -0.93 ± 0.75 | 0.40 ± 1.71 | -4.28 | 6.90E-05 | 1.92 |
| 3-ethyl-2,5-dimethyl-pyrazine | 11.88 | 13360-65-1 | NIST17 | -0.90 ± 1.77 | 0.38 ± 1.74 | -2.24 | 9.95E-04 | 1.86 |

^a^Compounds listed are those that met significance criteria of *Adj. P* < 0.001 and |log_2_ fold change| > 2.0

^b^Identification method:

S = Experimental spectrum matched to authentic standard

N17 = Experimental spectrum matched to NIST17 spectral library

MSI = Experimental spectrum and presumed structure matched to compound ID in MS Interpreter
